# N-Acetylaspartate Biomarker of Stroke Recovery: A Case Series Study

**Published:** 2021-03-31

**Authors:** Tyler Austin, Ali Bani-Ahmed, Mihaela Carmen Cirstea

**Affiliations:** 1 University of Missouri School of Medicine, Columbia, Missouri, USA; 2 Hoglund Brain Imaging Center, University of Kansas Medical Center, Kansas City, Kansas, USA; 3 Department of Physical Therapy & Rehabilitation Science, University of Kansas Medical Center, Kansas City, Kansas, USA; 4 Department of Neurology, University of Kansas Medical Center, Kansas City, Kansas, USA; 5 Department of Rehabilitation Sciences, Jordan University of Science and Technology, Ar-Ramtha, Jordan; 6 Department of Physical Medicine & Rehabilitation, University of Missouri, Columbia, Missouri, USA

**Keywords:** Chronic subcortical stroke, arm therapy, primary motor cortex, N-acetylaspartate

## Abstract

**Background and purpose:**

Strong experimental neurobehavioral evidence suggests that intensive training improves arm motor disability after stroke. Yet, we still have only limited understanding why some patients recover more completely and others do not. This is in part due to our limited knowledge of the neurobiological principles of recovery from stroke. Mounting evidence suggests that functional and structural remapping of the primary motor cortex (M1) plays a major role in arm recovery after stroke. We used MR Spectroscopy to test the hypothesis that therapy-related arm improvement is associated with changes in levels of a putative marker of neuronal integrity (N-acetylaspartate, NAA) in M1 controlling the paretic arm (ipsilesional M1) in chronic stroke patients (n=5).

**Methods:**

Patients (1 female, age, mean ± SD, 58.4 ± 5.8 years) underwent 4-week arm-focused motor training (1080 repetitions of a reach-to-grasp task) at 13.6 ± 5.3 months after stroke onset. NAA levels in the ipsilesional M1 and arm impairment (Fugl-Meyer, FM, 66=normal; proximal FM, FM_p_, 30=normal) were assessed prior to and immediately after training.

**Results:**

At baseline, patients exhibited moderate-to-mild arm impairment (FM, 47.2 ± 18.8, FM_p_, 22.2 ± 8.6) and showed lower levels of NAA compared with age/sex-matched healthy controls (10.2 ± 0.9 mM in patients *vs.* 11.6 ± 1.6 mM in controls, *p*=0.03). After training, arm impairment improved (FM by 7%, 50.6 ± 17.5, *p*=0.01; FMp, by 5%, 23.4 ± 8.2, *p*=0.2) and NAA levels increased by 10.5% (11.2 ± 1.2 mM, *p*=0.1). Changes in NAA positively correlated with changes in FM (*r*=0.63, *p*=0.2) and FM_p_ (*r*=0.93, *p*=0.03), suggesting that patients who show greater neuronal changes have a better chance of recovery.

**Conclusions:**

Our data suggest the potential use of M1 NAA as a biomarker of motor recovery after stroke. However, because of our small sample, these preliminary results should be interpreted cautiously. Further work with larger sample sizes is warranted.

## Introduction

Stroke frequently results in long-term neurological deficits that significantly interfere with daily activities and functioning [1]. Motor deficits are a large contributor to this disability; persistent arm impairment after stroke severely compromises patients’ quality of life [2,3]. Overall, these patients do exhibit some recovery of arm impairment early after stroke, but for reasons that remain unknown the extent of such recovery is highly variable between individuals. Given the higher prevalence of stroke in the elderly, the burden of stroke is likely to increase as our population ages. Moreover, the incidence of younger people suffering from this debilitating disease increased lately [4]. This is an issue of considerable impact for the U.S. healthcare system; there are at least 6 million stroke survivors [5] with 795,000 new cases joining this group every year [6]. The cost of related care is among the fastest-growing expenses for Medicare; the actual annual cost is $34 billion [6] with a projected cost of $240 billion by 2030 [7]. Therefore, minimizing motor disability is of high priority. Despite dozens of phase III clinical trials, current rehabilitation options are scarce and of limited effectiveness. This is in part due to our poor understanding of the neurobiological principles of recovery in humans. Here we applied for the first time in a cohort of chronic stroke survivors quantitative methods (MR Spectroscopy, MRS) to study whether changes in neuronal status of the motor cortex controlling the impaired arm parallel changes in clinical impairment during an arm-targeted therapy.

MRS is uniquely able to non-invasively obtain information about the neuronal status in the human living brain by means of biomarkers related to neuronal health, i.e., N-acetylaspartate [8]. We investigated in patients suffering from subcortical stroke whether there are indications of therapy-related changes of N-acetylaspartate (or NAA) in the paretic hand/arm territory in M1 (ipsilesional M1). Our previous studies have identified lower levels of NAA in uninjured, remote motor cortices, including M1, and such changes accurately related to poor arm function [9–11] and predicted arm recovery [12]. Since NAA was measured in uninjured brain areas where the neuronal death is likely to be minimal, we interpreted lower NAA as reflective of neuronal metabolic dysfunction rather than a marker of neuronal death [13]. To our knowledge, there exists only one published study investigating longitudinal changes in cortical NAA after stroke [14]. Glodzik-Sobanska et al. [14] found an increase in NAA levels in bilateral prefrontal cortices from 14 days to 100 days post-stroke in 27 patients with NAA in uninjured (contralesional) prefrontal cortex related to neurological improvement. This finding was interpreted as evidence for contralesional hemispheric involvement in the recovery process in subacute stroke. However, the question of whether such changes manifest during the chronic stage of stroke (defined here as more than 6 months post-onset) is unknown. Furthermore, it is possible that changes in ipsilesional NAA could have been overestimated by using ratio to creatine in Glodzik-Sobanska’s study. The reason for this is that creatine, a cellular energetics biomarker used as an internal reference against which NAA was compared, could be altered in the injured brain [15]. In the Glodzik-Sobanska et al. study, in some patients, the prefrontal cortex included injured brain tissue too. Nevertheless, the increase in NAA in this initial report is suggestive of possible cortical neuronal-level changes related to recovery from stroke and serves as an important motivation for the current study.

Here, we used MRS to assess in five chronic subcortical stroke survivors whether ipsilesional M1 NAA levels change along with changes in arm function during an arm therapy. On the basis of our aforementioned studies in subcortical stroke [9–12], we predicted that the baseline levels of NAA would be lower in patients relative to a matched healthy group (*n*=11). On the basis of the well-proven experience-dependent neuroplasticity after stroke [16–19] and the only longitudinal MRS-NAA study in stroke [14], we hypothesized that NAA levels would increase with therapy and such increase would parallel therapy-related arm improvement. Considering the potential role of the contralesional M1 in motor learning and recovery after stroke [20,21], we also explored changes in NAA in this area during therapy.

In addition to the primary hypotheses concerning NAA, we tested several secondary predictions involving four other metabolites: myo-inositol (mIn, considered a putative glial marker), glutamate-glutamine complex (Glx, a marker of glutamatergic neurotransmission), choline (Cho, a marker of cellular membrane turnover), and creatine (Cr, central energy marker of both neurons and astrocytes). In the ipsilesional M1, we expected practice would 1) increase mIn, based on the role of glia in supporting neuronal function (by modulating synaptic transmission, synaptic plasticity [22–24]and on the direct relationship between glial and synaptic responses in spared cortical areas after a focal brain injury [25]; 2) possible increase Glx, considering the effects of motor learning on cortical excitability [18,26,27]; and 3) no change Cho or Cr, based on the slim chance of altered cell membrane integrity or changes in brain energetics in this stage of stroke. For the reasons described for NAA above, we also explored the secondary metabolites changes of hand territory ipsilateral to the impaired hand (contralesional M1).

## Materials and Methods

The study was performed with the approval of the Kansas University Medical Center Human Subjects Review Board. All participants gave written informed consent before participation.

### Participants

Patients were selected from our stroke database between 2007 and 2011. Out of a 156 pool, 10 met the following inclusion criteria: a) a single subcortical infarction in the dominant (left) hemisphere (verified on T1/T2-weighted MR images), b) at least 6 months post-stroke, c) mild to severe arm motor impairment (Fugl-Meyer, FM [28], 66>score>10), d) no receptive aphasia (Token test), e) no visual attention deficits (Cancellation test) or apraxia (clinical observation of the use of scissors to cut paper), f) no uncontrolled depression (SIG-E-CAPS<“moderate” range), and g) able to participate in a 12-day therapy. Patients were excluded for any neurological, psychiatric, and/or orthopedic disease that interfered with training and/or data analysis, and for MRI contraindications. Two patients did not complete the protocol due to decline in health (unrelated to this study) and 2 were excluded from analysis due to MRS data contamination in the post evaluation. Thus, only 5 patients completed the experimental protocol (Table 1).

Eleven healthy controls with normal structural MRI and no history of neurological/psychiatric /orthopedic disease were also recruited.

### MR imaging and clinical assessments

Patients underwent MR imaging (3T Siemens Allegra MR system) and clinical assessments within 24h prior and immediately after 12-day therapy. Controls underwent one MR imaging.

**Figure 1.**
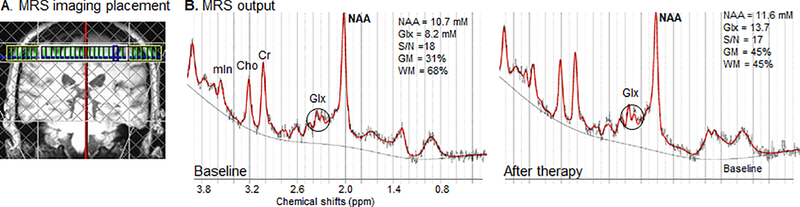
**(A)** MRS imaging slab (yellow rectangle) and saturation bands (grey lines) were positioned on T1-weighted MR image on the basis of the anatomical landmarks of the motor hand territory (omega shape) **(B)** MRS output (LCModel) from one MRS voxel located in left motor cortex in a stroke patient (S1, Table 1) shows distinct peaks corresponding to NAA (at 2.02 ppm), Glx (2.05–2.50 ppm), Cr (3.02 ppm), Cho (3.22 ppm), and mIn (3.56 ppm) at baseline (signal-to-noise ratio of 18) and after therapy (signal-to-noise ratio of 17). Cramér-Rao lower bands (CRB) were less than 15 for all neurochemicals in these voxels. ppm, parts per million; GM, grey matter in that voxel, WM, white matter in that voxel.

MR imaging protocol consisted of structural MRI and MRS and has been previously detailed by our group [12]. Briefly, a high resolution T1 MPRAGE (Magnetization Prepared Rapid Acquisition Gradient Echo; TR=2300 ms; TE=3 ms; field of view=240 mm; matrix=256 × 256; resolution=1 × 1 × 1 mm^3^) was used to quantify a) stroke volume (MedINRIA, Cedex, France; MIPAV, http://mipav.cit.nih.gov/), b) white matter hyperintensities (Fazekas visual rating scale [29]), and c) brain tissue composition in the spectroscopic voxels (SPM, Wellcome Trust Centre for Neuroimaging, University College London, UK). Intra-/inter-rater reliability of stroke volume and white matter hyperintensities measurements was verified. Immediately after the MPRAGE acquisition was completed, the volume of interest for MRS imaging was placed in the frontal and parietal lobes in both hemispheres, centered on the “hand knob” in M1, using standard sulcal and gyral landmarks [30] (Figure 1A). MRS was acquired using a point-resolved spectroscopy sequence (PRESS, TE=30 ms; TR=1500 ms; matrix size=16 × 16; field of view=160 mm; slice thickness=15 mm; in-plane resolution=5 × 5 mm^2^; spectral width=1200 Hz). To minimize lipid artifacts from the scalp, eight outer voxel suppression bands (thickness=30 mm) were placed around the volume of interest. Automatic and manual shimming were used to achieve full-width at half maximum of <20 Hz of the water signal from the entire excitation volume. Metabolites’ levels were calculated using LCModel [31] and a custom-designed MATLAB program (7.1; MathWorks, Natick, MA). Only the spectroscopic voxels corresponding to the hand/arm territory in each M1 with signal-to-noise ratio (SNR)>10, Cramer-Rao bounds<20% for each metabolite, and brain tissue volume>75% were included in the analysis [9]. Brain tissue-corrected levels of metabolites were averaged to provide a mean metabolite level per M1. Voxel relocalization method [32] was used to ensure that comparable anatomical regions are sampled in pre- and post-therapy scans. Extreme care was used to limit head movement (cushioning, providing music [33]).

FM, a valid and highly reliable clinical and research scale [34,35], was administered immediately before and after therapy.

### Arm therapy

**Figure 2.**
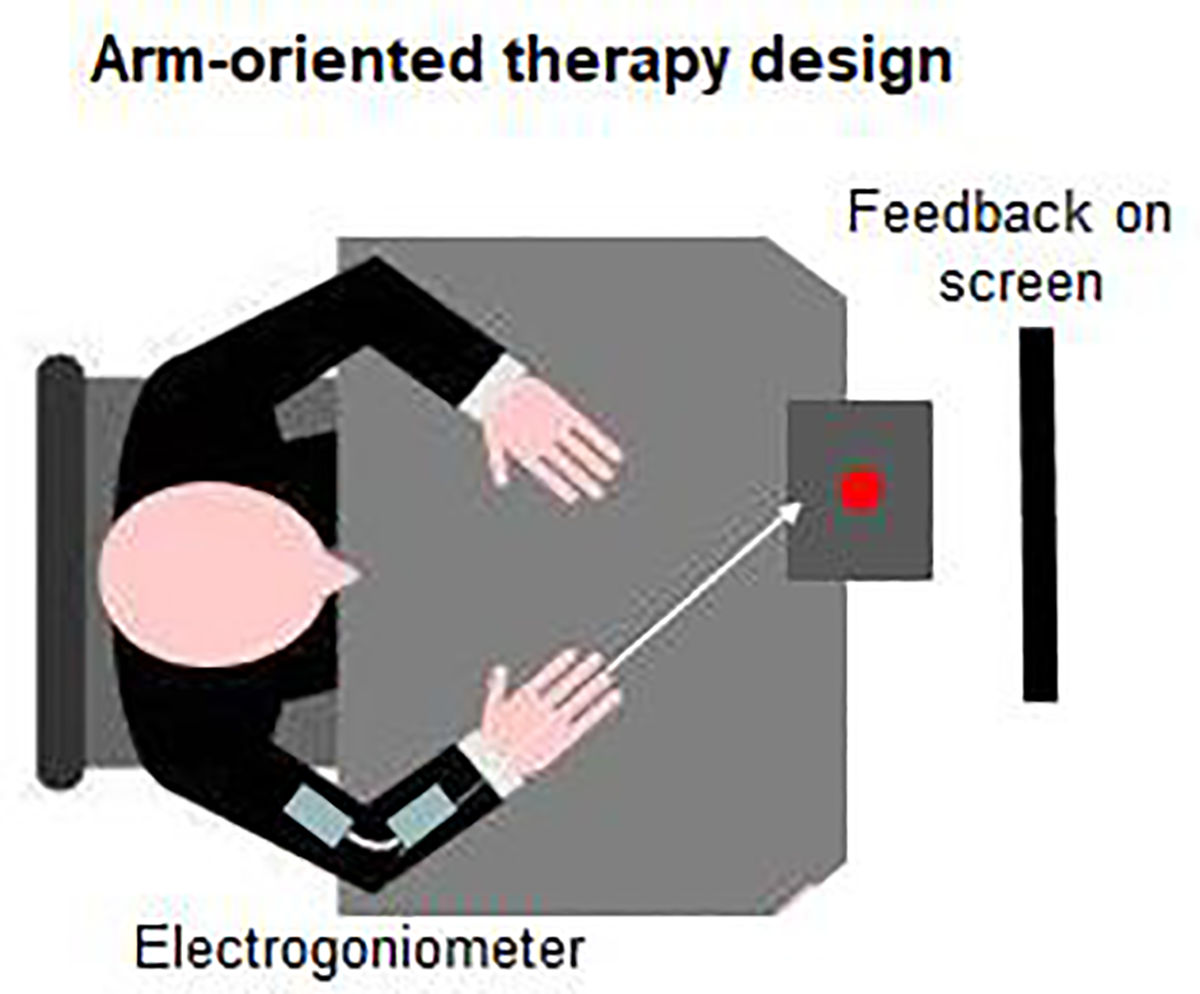
Arm therapy experimental design – top view. Patients were seated in front of a table and practiced a reach-to-grasp task for 90 times per day, 3 days per week, 4 weeks consecutively (*n*=1080 repetitions). During practice, attention was directed towards the elbow extension movement (feedback from the electrogoniometer was provided on a computer screen placed in front of patients). Patients had no arm or trunk restrictions during practice. Red circle signifies the object to be grasped.

This therapy is previously detailed [12]. Briefly, it consisted of 12 sessions of motor practice over a 4-week period (90 repetitions/session, a total of 1080 repetitions). Patients performed a high ecological reach-to-grasp task with the impaired hand. There were no arm or trunk restrictions during practice. Patients received visual feedback about their elbow extension movement (electrogoniometer, Exos, Inc., Woburn, MA, USA, Figure 2), an altered movement component after stroke well-studied in our lab [36]. To minimize dependence on feedback, the feedback frequency was decreased throughout the practice session (for the first 30 trials, feedback was provided every trial, for the second 30 trials, every second trial, and for the last 30 trials, every fifth trial). All participants completed the training sessions and received the same feedback during the practice. Because the therapy focus was on elbow movement, we also included in our analysis the FM components specifically dealing with elbow (and shoulder) joint movements (proximal FM, FM_p_, normal=30).

### Statistical Analysis

Patient characteristics were summarized by frequencies and proportions for qualitative and by mean (SD) for quantitative variables. *t*-test was employed to assess a) between-group baseline metabolites (Kolomogorov-Smirnov normality tests revealed that NAA and other metabolites were normally distributed in stroke and control groups at baseline, *p*>0.05), b) therapy effects on NAA (other metabolites) and FM/FM_p_ in the stroke group, and c) the reliability of infarct volume and white matter hyperintensities measurements. Pearson rank order correlation was used to determine the relationships between therapy-related changes in NAA (and secondary metabolites) levels (e.g., ΔNAA) with those in FM (ΔFM, ΔFM_p_). Significant level was considered at *p*<0.05 (SPSS 23.0; SPSS Inc, Chicago, IL).

## Results

### Baseline demographics, medical history, MRI findings, and arm function

Five right-handed survivors (80% male, age, mean ± SD, 58.4 ± 5.8 years old) suffering from a single infarct (volume, 13.5 ± 11.7 cm^3^) located at the subcortical level in the left hemisphere and with no to mild white matter hyperintensities (Fazekas scores varied between 0 and 1), completed the entire protocol at 13.6 ± 5.3 months post-stroke (Table 1). Patients were not apraxic, cognitively impaired, or uncontrolled depressed, and they were not receiving current inpatient/outpatient treatment or spasticity medication. At baseline, patients presented with moderate-to-mild arm impairment (FM, 47.2 ± 18.8, FMp, 22.2 ± 8.6) and were on anti-hypertensive (100%), anti-platelet (80%), and/or cholesterol-lowering (60%) medication. Control group (73% male, 53.5 ± 14.5 years old, without pathological white matter lesions (Fazekas=0), 73% on anti-hypertensive medication and cholesterol lowering) provided an adequate comparison group with no differences in potential confounding demographic influences: age, sex, scholar years (*p*>0.05 for all).

A high agreement regarding infarct volume (Wilcoxon rank sum test, *p*<0.01 for both intra- and inter-rater analyses) and white matter hyperintensities (paired *t*-test, *p*<0.01 for both) measures was found between 2 evaluators.

### MRS quality and analysis (Table 2)

Brain tissue (grey matter and white matter) composition in the spectroscopic voxels were similar between groups and hemispheres at baseline and after therapy (Table 2). Likewise, MRS data quality was similar between groups at baseline and after therapy. A high spectral resolution was detected in all participants for each hemisphere (SNR>10, Table 2) and a full width at half maximum of the point spread function of all metabolites <20 Hz for all spectra (Figure 1B). Therefore, the differences in metabolites levels presented below are not due to regional structural or spectral resolution differences.

### Baseline NAA (and secondary metabolites, Table 3) levels

As predicted, relative to controls, patients showed lower NAA in the ipsilesional M1 compared to the homologues area in controls (10.2 ± 0.9 mM *vs.* 11.6 ± 1.6 mM, *p*=0.03). NAA levels in the contralesional M1 tended to be lower but did not differ significantly from those detected in controls (10.4 ± 1.3 *vs.* 11.6 ± 1.8, *p*=0.2).

We failed to detect differences between patients and controls in mIn, Glx, Cho, or Cr in either the ipsilesional or contralesional M1 (Table 3).

### Therapy effects on arm impairment (Figure 3A)

Similar to findings reported from studies involving similar training protocol in a similar stroke sample [37], patients showed significant clinical gain after training (FM after therapy, 50.6 ± 17.5, an increase of 3.4 ± 1.7, *p*=0.01). Lastly, as expected, due to our type of training, FM_p_ also improved by 1.2 ± 1.8, though was not statistically significant (*p*=0.2).

### Therapy effects on NAA (and secondary metabolites) levels (Figure 3B)

Consistent with our prediction, ipsilesional NAA increased from 10.2 ± 0.9 to 11.2 ± 1.2 after therapy, but without reaching statistical significance (*p*=0.1). Notably, baseline between-group differences in ipsilesional NAA receded after therapy (*p*=0.6). Contrary to our hypothesis that therapy would also impact the contralesional M1 neuronal status, NAA did not change with therapy (from 10.4 ± 1.3 at baseline to 10.3 ± 1.9 mM after therapy, p=0.9). This may suggest that ipsilesional changes are favored over contralesional changes during this type of therapy and in this sample.

Patients also exhibited a tendency toward increased ipsilesional Glx and no significant change in contralesional Glx. Patients displayed no significant changes in ipsi- or contralesional mIn, Cho, and Cr (Table 3). As predicted, ipsilesional Glx increased from 10.3 ± 2.1 mM to 12.7 ± 2.6 mM with therapy, although without reaching statistical significance (*p*=0.08). This result may reflect the plastic changes induced by motor learning in M1 [18,26,27]. Contrary to our hypothesis that ipsilesional mIn would change with therapy, the mIn levels did not change significantly with therapy in this sample (*p*=0.5). As predicted, ipsilesional Cho and Cr levels did not change with therapy (*p*>0.05 for both). Similar to contralesional NAA, no significant changes were found for the secondary metabolites in the contralesional M1 (*p*>0.05 for all).

### Relationships between NAA (and secondary metabolites) changes and clinical gain (Figure 4)

As predicted, therapy-related changes in ipsilesional NAA were positively correlated with changes in FM (*r*=0.63, *p*=0.2) and FM_p_ (*r*=0.93, *p*=0.03). These results suggest that local changes in ipsilesional NAA levels may provide a means of assessing functionally-relevant neuroplastic changes during this therapy. Although contralesional changes in NAA levels were minimal, we explored their relationships with clinical gain and found no significant relationships (FM, *p*=0.5; FM_p_, *p*=0.9). This finding reinforces the theory of ipsilesional over contralesional recruitment during this type of training.

Contrary to our prediction, therapy-related changes in ipsilesional Glx were negatively correlated with clinical changes (FM, *r*=−0.89, *p*=0.04; FM_p_, *r*=−0.47, *p*=0.4). Similar to the contralesional NAA, there were no significant relationships between the changes in the contralesional Glx and those in clinical scores (FM, *p*=0.6; FM_p_, *p*=0.8).

## Discussion

To our knowledge, this is the first study showing that NAA levels in the ipsilesional M1 increase along with arm improvement in a sample of chronic subcortical stroke survivors. This study had 3 main results. First, our patients, although chronic, displayed a significant improvement in arm impairment with therapy. Second, NAA levels showed a trend toward increase with therapy; the statistically significant baseline between-group difference in NAA levels receded after therapy. Third, therapy-related NAA increase positively correlated with arm improvement. These will be discussed in greater detail below.

### Therapy improved arm impairment

**Figure 3.**
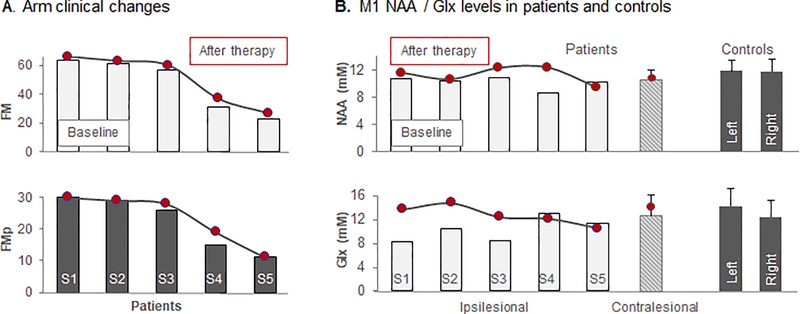
**(A)** Changes in arm clinical scores (FM, grey columns; FMp, black columns) from baseline (column) to post-therapy (circle) in individual stroke patient (S1 to S5). **(B)** Changes in NAA (upper row) and Glx (lower row) levels in ipsilesional (for individual patient, S1 to S5) and contralesional (mean value for all patients) M1 from baseline (columns) to post-therapy (circle). NAA and Glx levels in left and right M1 in controls are also presented (dark columns).

Our patients saw a clinically and statistically significant gain in arm function as measured by FM (Figure 3A). In addition to clinical improvement, our patients subjectively noticed improvements in activities of daily living, i.e., able to use a deodorant after therapy. Certainly, clinical changes were important, but these subjectively described improvements are worth emphasizing as they indicate the potential of this training paradigm to generalize to other behaviours that are of high ecological relevance. These results further support the potential of these patients to improve beyond the acute stage well into the chronic stage (as far as 20 months post-stroke) of stroke.

### There was a trend toward increased levels of ipsilesional NAA with therapy that positively correlated with clinical gain

**Figure 4.**
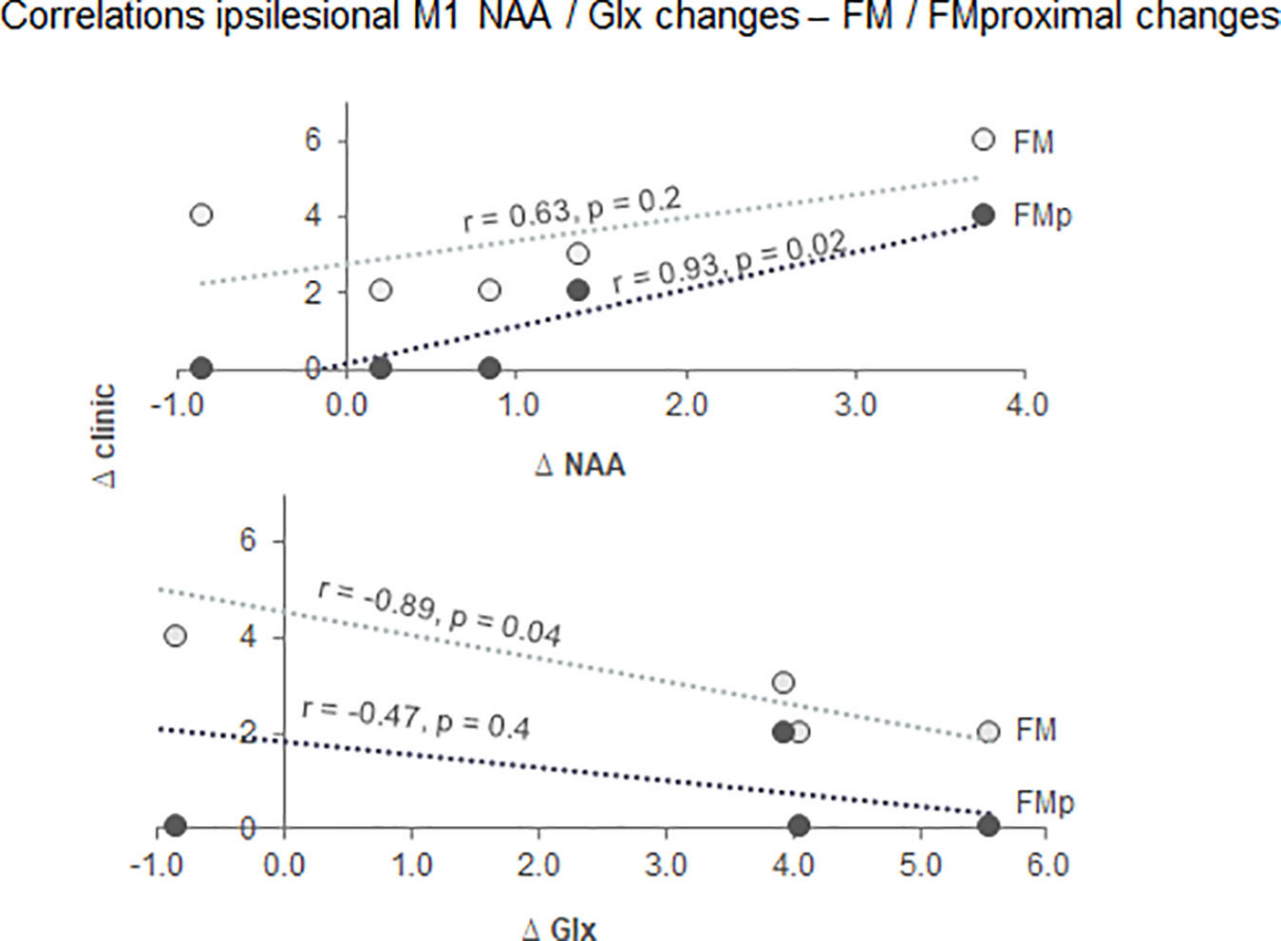
Pearson correlations (r, p) between changes in ipsilesional NAA (Δ NAA, upper row) and Glx (Δ Glx, lower row) levels and changes in clinic scores (Δ FM, grey circles; Δ FMproximal or FMp, black circles).

To our knowledge, we are the first to evaluate therapy-related changes in ipsilesional M1 levels of NAA in patients suffering from chronic stroke. Patients displayed lower NAA levels at baseline that increased with therapy (Figure 3B), although without reaching statistical significance (*p*=0.1). In line with prior reports demonstrating metabolic cell body changes after axonal injury [38] (all our patients have the infarct located at the subcortical level) and/or metabolic abnormalities associated to post-stroke diaschisis [39], baseline lower levels of NAA are presumably reflecting neuronal metabolic depression [9–11]. We speculate that an increase in NAA levels may echo the effects of therapy at the cellular level resulting in plastic changes in metabolically depressed neurons. For instance, an increase in dendritic arborization [40] is likely to yield an increase in NAA levels. Such changes would enable the neurons to i) recruit adjacent neurons with intact axons which potentially have muscle projections similar to that of metabolically depressed neurons, and/or ii) facilitate the activation of multiple novel muscle synergies that are required for motor skill acquisition. This may explain, in part of course, the arm improvement reported here. The positive relationship between NAA increase and arm improvement (Figure 4 upper row) further supports our interpretation. While the NAA increase was not significant, likely due to the small sample size, the potential of a therapy in improving neuronal status of the ipsilesional M1 is promising and encouraging for further tailoring therapy based on such quantitative measures. However, further work with larger sample sizes is warranted.

### There was a trend toward increased levels of ipsilesional Glx that negatively correlated with clinical gains; there were no significant changes in or correlation between ipsilesional Cho or Cr and clinical gains

Our preliminary data showed that therapy also induced an increase, although nonsignificant statistically (*p*=0.08, Figure 3B), in ipsilesional levels of Glx. This confirmed our prediction and further demonstrated the use dependent plasticity of the intact M1 in chronic stroke. Put differently, an increase in M1 excitability may occur when the same neural networks are repeatedly used [18,26,27]. Yet, Strangely we observed a negative relationship between such increase and performance improvement (Figure 4 lower row). Many prior studies indicated the M1 plays a critical role in performance improvement (see above). We are not able to explain well this relationship; yet, we observed that Glx increased only in the mildly impaired patients (S1–3, Table 1) and decreased in the moderately impaired patients (S4–5, Table 3, Figure 3B lower row), thus, combining all patients may, at least in part, explain such a relationship. However, because of our small sample, this preliminary result should be interpreted cautiously.

### Therapy did not alter significantly levels of contralesional NAA or secondary metabolites; there were no significant relationships between contralesional metabolite changes and clinical gains

Our results failed to support our hypothesis that contralesional M1 contributes to arm improvement during this therapy. This is somewhat disappointing because we previously found in a larger sample of patients a significant relationship between NAA levels in contralesional M1 and proximal (shoulder/elbow) impairment [11], suggestive of a potential role of this area in recovery from such impairment. However, in addition to limited size, our sample did not include severely impaired patients, in which the contralesional M1 is considered a viable alternative to control the impaired arm [20,21], and in which such metabolic changes and correlations would be expected.

## Study limitations

The biggest limitation of this study is its small sample size (*n*=5). However, the patient group was homogenous due to our restrictive inclusion criteria. Another limitation of this study is its generalizability. Our patient population consisted of chronic moderate to mild subcortical stroke survivors with only left hemisphere injured. The results of this study may not apply to right hemispheric acute or subacute stroke or to those with more severe arm impairment. Additionally, our study focused on metabolic level of M1, preventing us from being able to comment on the involvement of other brain regions, such as the premotor cortex [41], in clinical improvement reported here.

## Conclusions

Although this is a case studies study, we hope that these data will act as a starting point for further research to test the potential of NAA measures to provide a means of assessing neuroplastic changes in spared brain areas and possibly a biomarker of therapy response in the stroke population. The results of this study suggest that motor therapy in the chronic stage of stroke, combined with NAA measurements, are encouraging for the possibility of making therapeutic accommodations for future patients based on NAA levels, in addition to other clinically important factors. Further research and understanding in this area will undoubtedly have major implications for rehabilitation of these patients, for which we have little to offer.

## Figures and Tables

**Table 1.** Demographic and clinical characteristics of stroke survivors.

Patient	Age/sex	Months after onset	Infarct location	Baseline FM	Global grey matter^a^	Global white matter^a^	Infarct volume^a^/^b^	Fazekas^c^
**1**	57/M	6	L/BG, CR	64	876.6	671.8	25.3/1.6	0
**2**	49/M	20	L/PLIC, BG	61	893.2	708.2	17.9/1.2	0
**3**	60/M	15	L/P	57	799–3	651.2	1.2/0.08	1
**4**	63/F	11	L/P	31	809.9	643.8	0.7/0.05	1
**5**	63/M	16	L/PLIC, BG	23	665.2	725.7	22.5/1.6	1

a =cm^3^

b =% infarcted brain volume/total brain volume

c 0=normal, ^c^1=normal in aged population

M: Male; F: Female; L: Left; FM: Fugl-Meyer, normal=66; BG: Basal Ganglia; CR: Corona Radiata; PLIC: Posterior Limb Internal Capsule; P: Pons

**Table 2.** MRS quality: signal-to-noise ratio (SNR) and brain tissue composition (%) in Each M1 at baseline and after therapy in the stroke group compared to the control group.

M1/groups	SNR		Brain tissue composition	
**Ipsilesional M1 in stroke**				
	Baseline	After	Baseline	After
**Mean ± SD**	11.4 ± 0.5	11.6 ± 1.1	87.9 ± 5.6	85.8 ± 6.8
***p*** ^a^	*0.6*		*0.6*	
**Left M1 in control**				
**Mean ± SD**	12.5 ± 2.6		89.7 ± 7.3	
***p*** ^b^	*0.2*	*0.4*	*0.6*	*0.3*
**Contralesional M1 in stroke**				
	Baseline	After	Baseline	After
**Mean ± SD**	11.3 ± 1.1	11.2 ± 1.1	84.4 ± 9.4	91.2 ± 5.2
***p*** ^a^	*0.8*		*0.2*	
**Right M1 in control**				
**Mean ± SD**	12.1 ± 2.2		85.6 ± 4.9	
***p*** ^b^	*0.4*	*0.3*	*0.8*	*0.1*

a t-test after therapy *vs.* baseline within the stroke group

b t-test stroke *vs.* control groups (ipsilesional M1 *vs.* left M1; contralesional M1 *vs.* right M1 at baseline and after therapy)

**Table 3.** Secondary metabolites levels (mM) in patients (individual levels) and controls (mean ± SD for group).

M1/Groups	mIn		Glx		Cho		Cr	
**Ipsilesional M1 in stroke**								
**Patient #**	Baseline	After	Baseline	After	Baseline	After	Baseline	After
**1**	3–5	3.5	8.1	13.7	1.6	1.9	6.5	6.6
**2**	6.3	5.8	10.5	14.6	2.2	1.8	7.9	7.9
**3**	5–5	8.8	8.5	12.4	1.9	2.8	6.4	9.7
**4**	6.1	8.4	13.1	12.1	2.0	2.2	8.8	10.6
**5**	5–3	4.2	11.3	10.5	2.1	1.5	7.9	6.4
**Mean ± SD**	5.3 ± 1.1	6.2 ± 2.4	10.3 ± 2.1	12.7 ± 1.6	2.0 ± 0.2	2.1 ± 0.5	7.5 ± 1.0	8.2 ± 1.9
***p*** ^a^	0.5		0.08		0.7		0.5	
**Left M1 in control**								
**Mean ± SD**	4.8 ± 0.9		12.2 ± 2.6		2.0 ± 0.5		8.1 ± 1.4	
***p*** ^b^	0.4	0.3	0.2	0.7	0.8	0.9	0.4	0.9
**Contralesional M1 in stroke**								
**Patient #**	Baseline	After	Baseline	After	Baseline	After	Baseline	After
**1**	4.0	4.0	8.3	16.3	1.5	1.2	6.1	5.7
**2**	5.9	4.7	9.5	13.1	1.9	1.7	6.8	7.2
**3**	6.1	4.5	16.2	11.6	1.9	2.1	8.6	8.7
**4**	6.1	7.3	10.3	11.9	2.0	2.4	8.6	10.5
**5**	5.2	5.2	12.1	7.1	1.7	1.4	7.2	6.5
**Mean ± SD**	5.3 ± 1.1	5.1 ± 1.3	11.3 ± 3.1	12.0 ± 3.3	1.8 ± 0.2	1.8 ± 0.5	7.5 ± 1.1	7.7 ± 1.9
***p*** ^a^	0.6		0.7		0.8		0.8	
**Right M1 in controls**								
**Mean ± SD**	5.1 ± 1.3		10.8 ± 2.5		2.1 ± 0.5		8.0 ± 1.0	
***p*** ^b^	0.5	0.9	0.7	0.5	0.09	0.2	0.4	0.8

a t-test after therapy *vs.* baseline within the stroke group;

b t-test stroke vs. control groups (ipsilesional M1 *vs.* left M1; contralesional M1 *vs.* right M1 at baseline and after therapy)
